# Supplementary material for: Linker histone H1 represses H3 tail acetylation induced by H4 tail acetylation and alters its dynamics
Source: Commun Biol. 2026 Apr 9;9:496. doi: 10.1038/s42003-026-09926-y (PMC13066561; doi:10.1038/s42003-026-09926-y)
Supplement: Supplementary file 2 — Supporting information [file 42003_2026_9926_MOESM2_ESM.pdf]

Supporting information for
Linker histone H1 represses H3 tail acetylation induced by H4 tail acetylation and alters its dynamics.

Ayako Furukawa[1,2], Kenta Echigoya[3], Samuel Blazquez[4,5], Masatoshi Wakamori[6], Hideaki Ohtomo[1], Yasuo Tsunaka[1], Takashi Umehara[6,7], Tsuyoshi Terakawa[4], Yoshimasa Takizawa[3,8], Hitoshi Kurumizaka[3,9,10] and Yoshifumi Nishimura[1*]

* Corresponding author: Yoshifumi Nishimura
Email: nisimura@yokohama-cu.ac.jp

This PDF file includes:

Supporting Figures S1 to S7

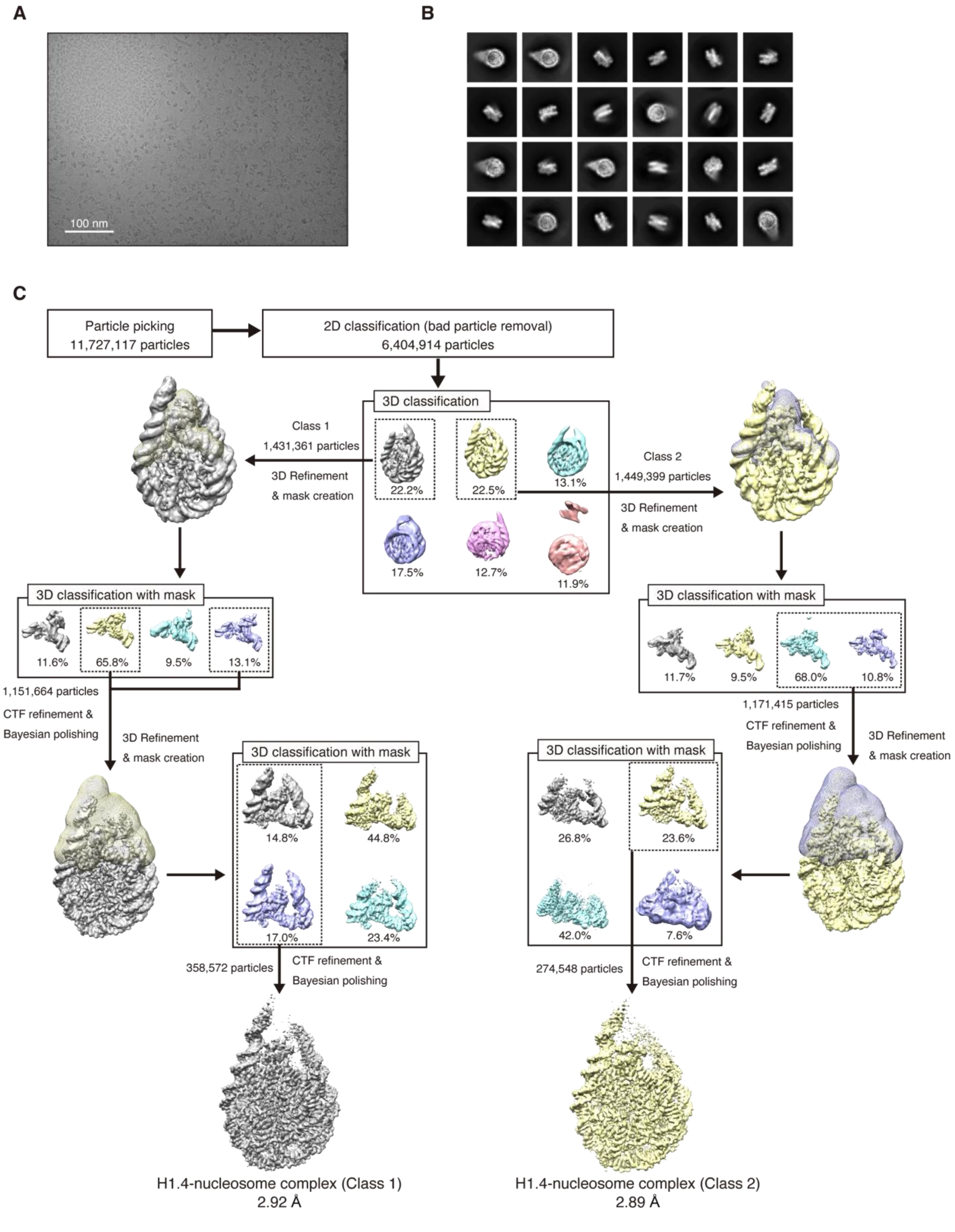

Fig. S1. Cryo-EM structure determination of histone H1.4 protein in complex with the reconstituted H4-4Kac nucleosome. A) Representative cryo-EM micrograph. **B**) Representative 2D class averages. **C**) Flowchart of cryo-EM image processing.

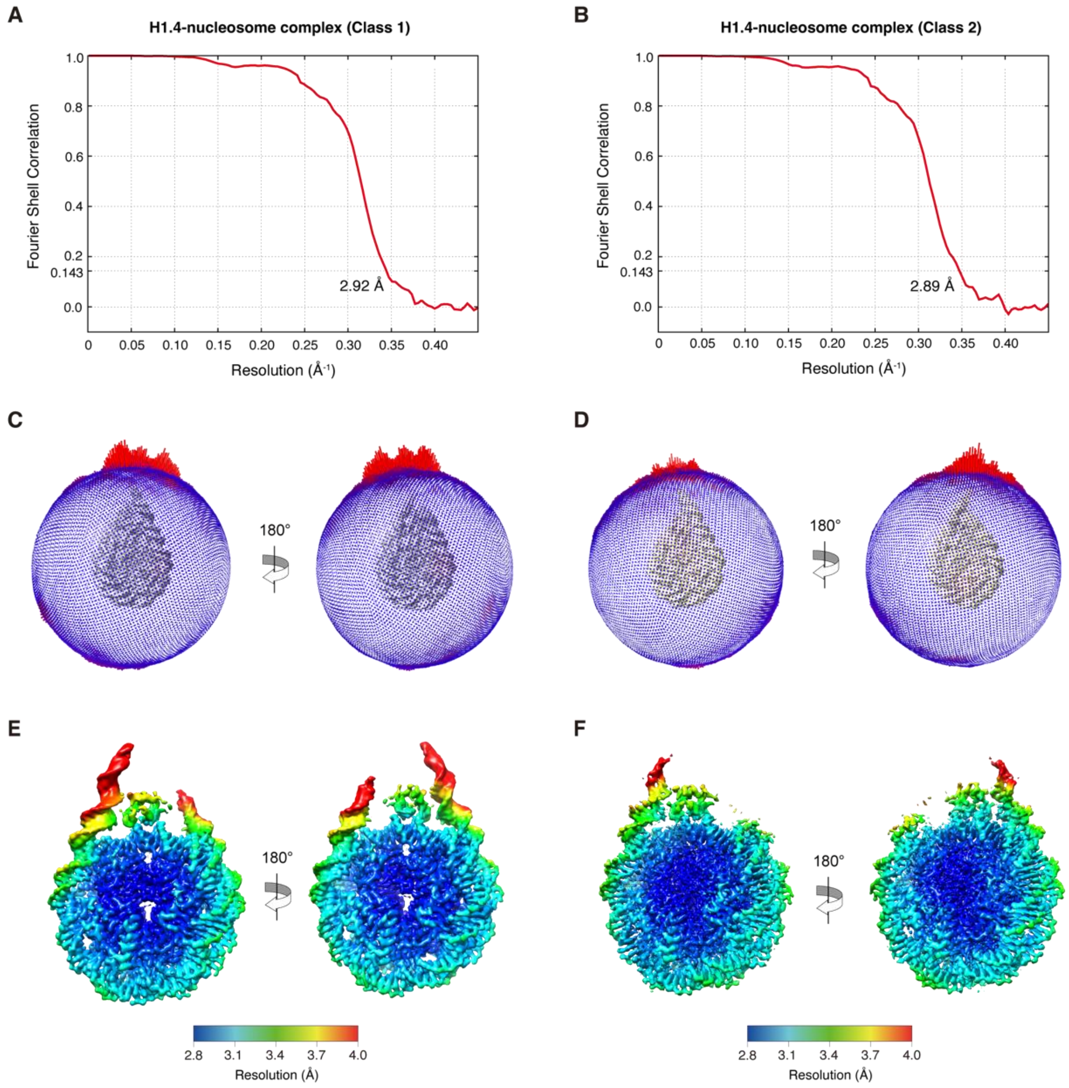

Fig. S2. Cryo-EM structure determination of histone H1.4 in complex with the reconstituted H4-4Kac nucleosome. A) Fourier-shell correlation curves of the Class 1 cryo-EM reconstruction. **B**) Fourier-shell correlation curves of the Class 2 cryo-EM reconstruction. **C**) Angular distribution of particle projections of the Class 1 cryo-EM reconstruction. **D**) Angular distribution of particle projections of the Class 2 cryo-EM reconstruction. **E**) Local resolution (Å) displayed on the sharpened full map of the Class 1 structure. **F**) Local resolution (Å) displayed on the sharpened full map of the Class 2 structure.

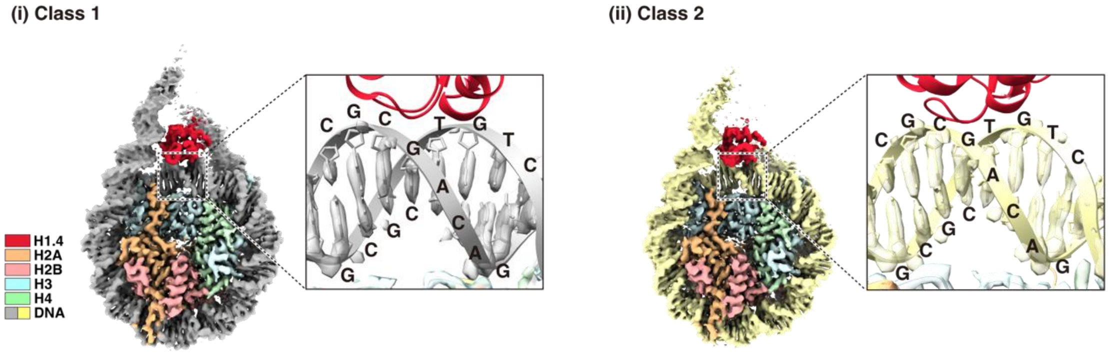

Fig. S3. Orientations of DNA in the two H4-4Kac chromatosome structures. DNA sequences were determined from cryo-EM maps of Class 1 (left) and Class 2 (right) H4-4Kac chromatosomes. Each panel shows close-up views of the cryo-EM maps around the nucleosomal dyad DNA together with the fitted model (PDB ID: 7K5Y).

A

Heat maps and percentage of contacts of the H3 N-tail in the acetylated nucleosome.

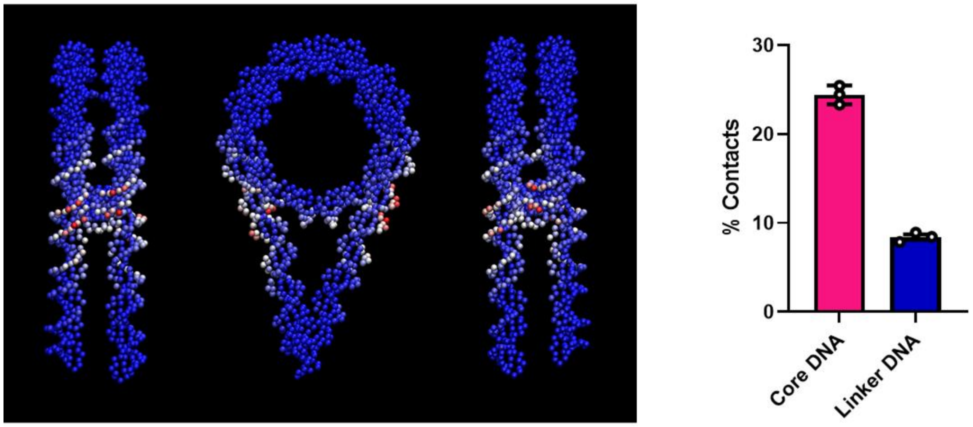

B

Heat maps difference between conventional and acetylated nucleosomes.

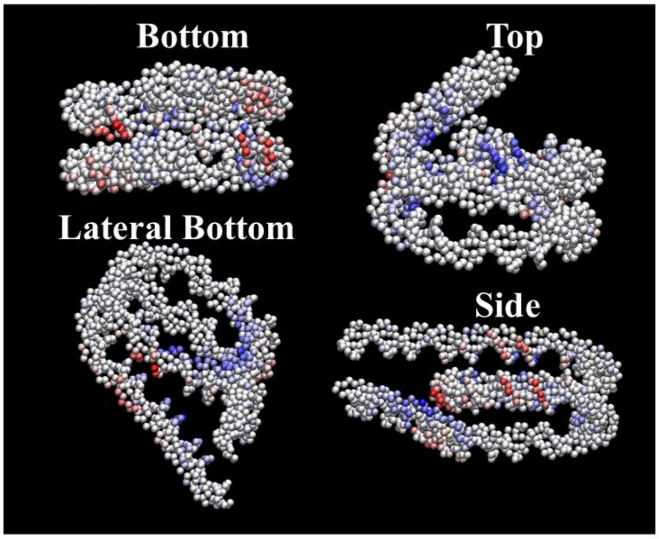

C

H4 N-tail and H3 N-tail contacts with DNA in conventional and acetylated nucleosomes.

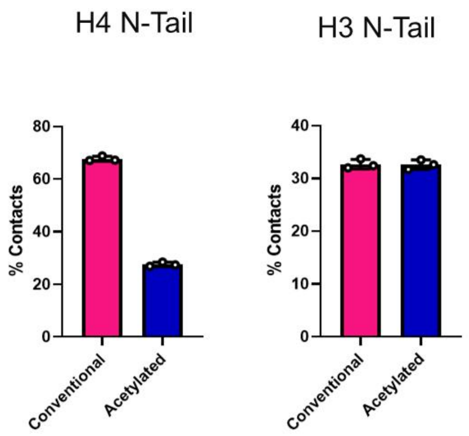

Fig. S4. Nucleosome molecular dynamics simulations. A) Heat maps of H3 N-tail contacts with DNA in the acetylated nucleosome (left) and percentage of contacts of H3 N-tail with core- and linker-DNA (right). **B)** Difference heat maps between the conventional and acetylated nucleosome. **C)** Percentage of contacts between the H4 N-tail or H3 N-tail and DNA in both the conventional and acetylated nucleosome in 10-μs simulations. Three independent MD simulations were performed for each state (n = 3) and error bars are defined as the standard deviation of the 3 independent simulations.

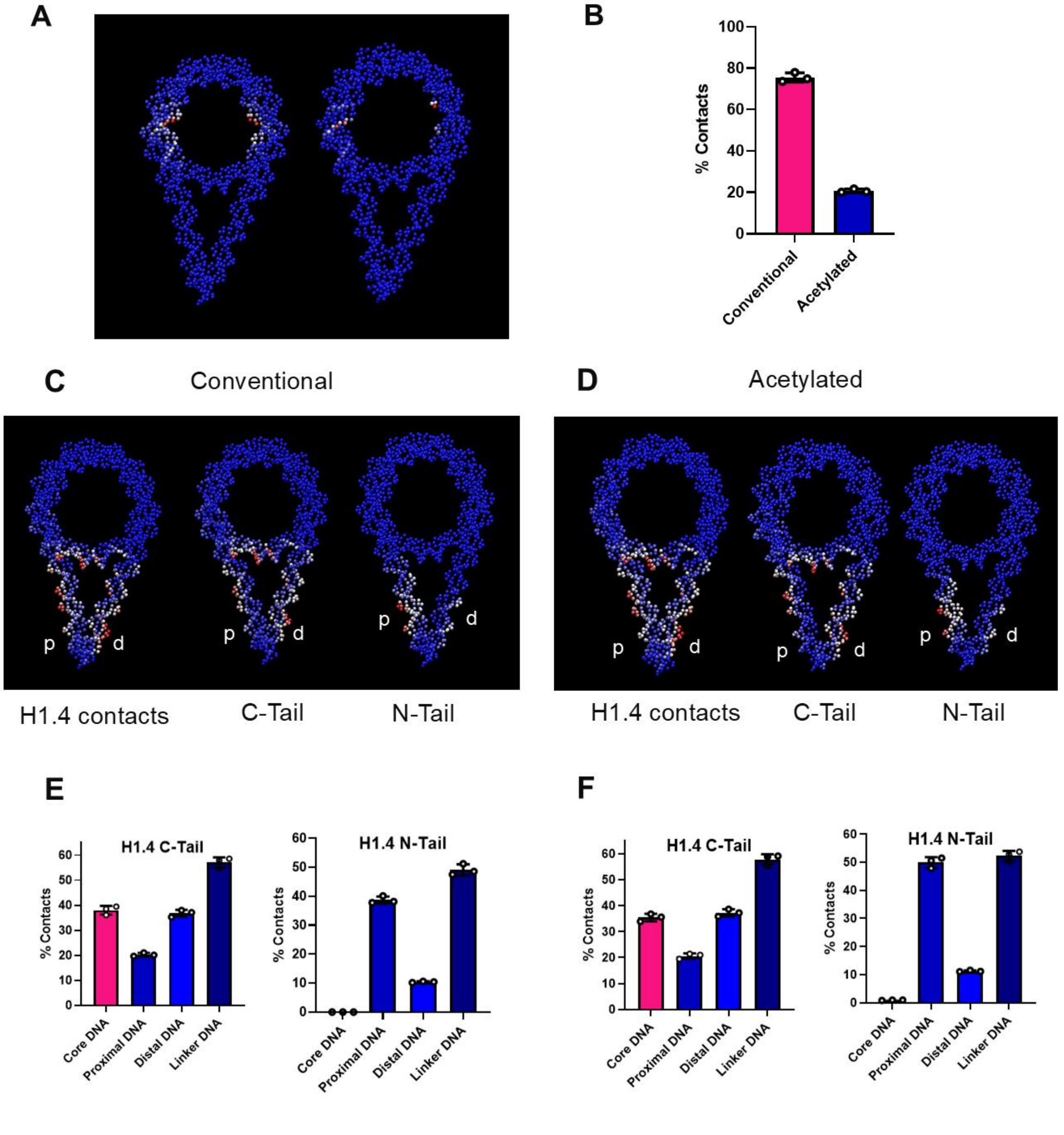

Fig. S5. Chromatosome molecular dynamics simulations. A) Heat maps of H4 N-tail contacts with DNA in the conventional (left) and acetylated (right) chromatosome. **B)** Percentage of contacts between the H4 N-tail and DNA. **C, D)** Heat maps of H1.4 linker histone with core-DNA, proximal ('p') and distal ('d') linker-DAN in the conventional (**C**) and acetylated (**D**) chromatosome. Left: total heat maps of contacts of H1.4. Center: heat maps of contacts of the H1.4 C-tail. Right: heat maps of contacts of the H1.4 N-tail. **E, F)** Percentage of contacts between the H1.4 C-tail (left) or N-tail (right) and core-DNA or linker DNA in the conventional (**E**) and actylated (**F**) chromatosome during10-μs simulations. Three independent MD simulations were performed for each state (n = 3) and error bars are defined as the standard deviation of the 3 independent simulations.

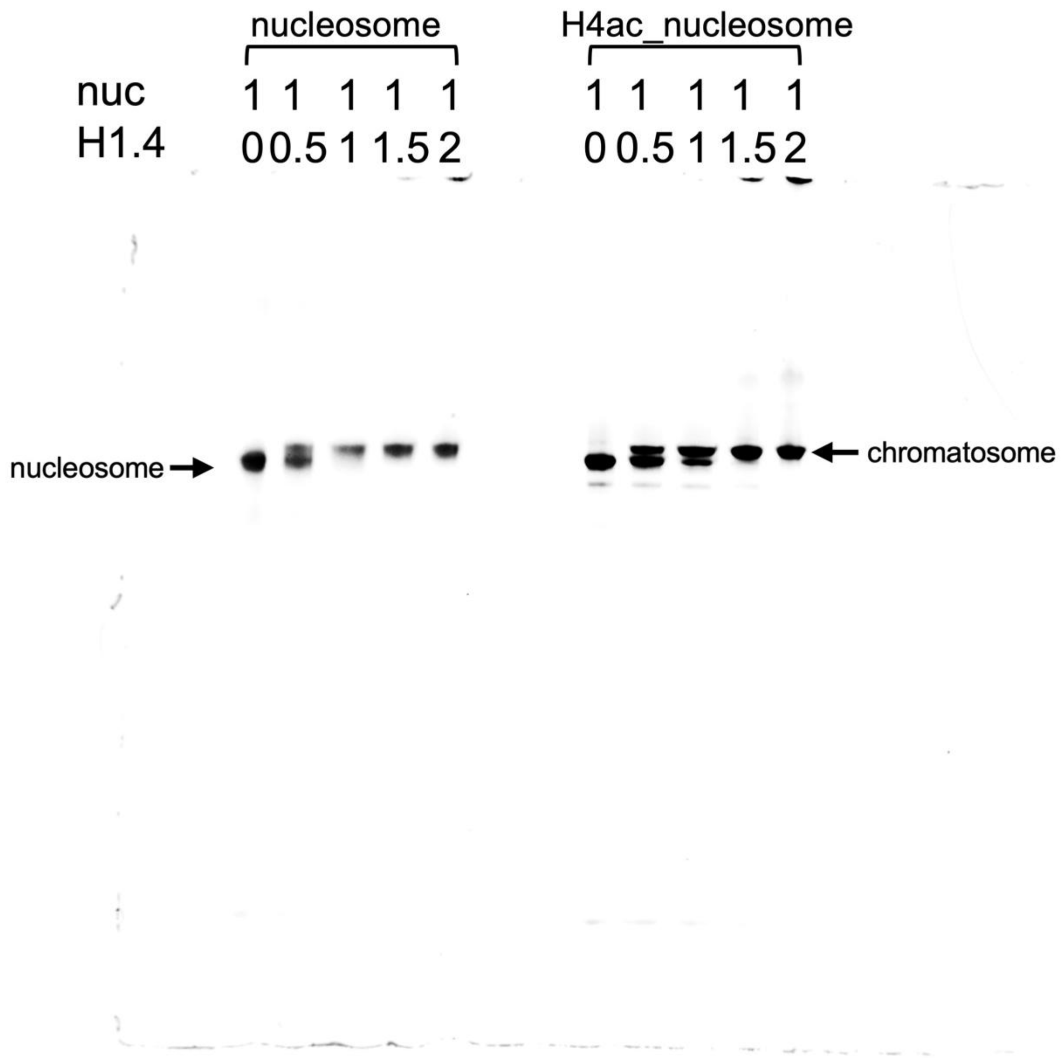

Fig. S6. DNA electrophoresis mobility shift assay of linker histone H1.4 binding to the nucleosome or H4-4Kac nucleosome. Nucleosomes with linker histone H1.4 added in ratios of 0.5, 1.0, 1.5, and 2.0 were subjected to electrophoresis at 4 °C on a 7.5% native-PAGE in 1 × Tris-glycine buffer and were visualized by SYBR Gold nucleic acid gel stain.

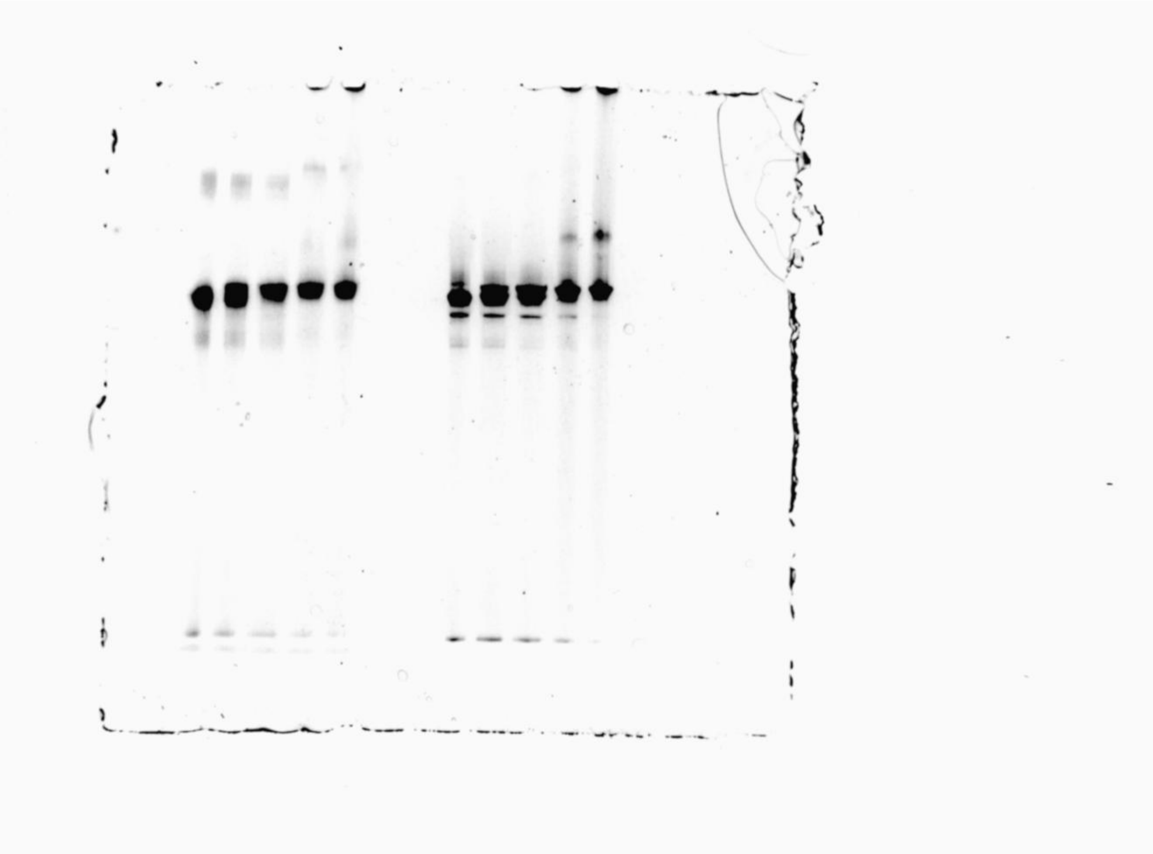

Fig. S7 Uncropped and unedited gel image of Fig. S6.
