# Supplementary material for: Linker histone H1 represses H3 tail acetylation induced by H4 tail acetylation and alters its dynamics
Source: Commun Biol. 2026 Apr 9;9:496. doi: 10.1038/s42003-026-09926-y (PMC13066561; doi:10.1038/s42003-026-09926-y)
Supplement: Supplementary file 3 — Description of Additional Supplementary Files [file 42003_2026_9926_MOESM3_ESM.pdf]

Description of Additional Supplementary Files

File name: Supplementary Data 1
Description: Dataset for Fig. 2A, 2C, 2D, S4A, S4C, S5B, S5E, and S5F.

File name: Supplementary Data 2
Description: Dataset for Fig. 3C.

File name: Supplementary Data 3
Description: Dataset for Fig. 4B.
